# Early postoperative CRP predicts major complications following cytoreductive surgery (CRS) and hyperthermic intraperitoneal chemotherapy (HIPEC)

**DOI:** 10.1515/pp-2022-0203

**Published:** 2023-06-02

**Authors:** Akash Kartik, Catharina Müller, Miklos Acs, Pompiliu Piso, Patrick Starlinger, Thomas Bachleitner-Hofmann, Travis E. Grotz

**Affiliations:** Mayo Clinic, Rochester, MN, USA; Medical University of Vienna, Vienna, Austria; Barmherzige Brüder Regensburg, Regensburg, Germany

**Keywords:** C-reactive protein (CRP), cytoreduction, heated intraperitoneal chemotherapy (HIPEC), peritoneal metastasis, postoperative complications

## Abstract

**Objectives:**

Cytoreductive surgery (CRS) and heated intraperitoneal chemotherapy (HIPEC) is associated with significant postoperative complications. Early detection of at-risk patients may lead to improved outcomes. The role of C-reactive protein (CRP) in predicting postoperative complications has only been recently investigated.

**Methods:**

Postoperative complications were categorized according to Clavien-Dindo classification and further divided into minor (Grade <3) and major complications (Grade ≥3A). Absolute CRP counts (mg/L) on postoperative days (POD) 1–7, and proportional change in CRP was compared and the area under (AUC) receiver operating characteristics (ROC) curve was calculated. Univariate and multivariate analysis was performed. Significant findings were externally validated.

**Results:**

Twenty-five percent of patients experienced one or more major complications. A CRP level of ≥106 mg/L on POD 2 and 65.5 mg/L on POD 4 were significantly associated with an increased risk of major complications with an AUC of 0.658 and 0.672, respectively. The proportional increase in CRP between POD 1 and 4 (ΔCRP POD 1/4) at a cut-off of 30 % had the best AUC of 0.744 and was the only independent risk factor for major complications (p<0.0001) on multivariate analysis. ∆CRP had an AUC of 0.716 (p=0.002) when validated in an independent database.

**Conclusions:**

CRP can be used in a variety of ways to predict major complications after CRS and HIPEC. However, the ∆CRP POD 1/4>30 % is the best indicator of major complications. Serial CRP measurements in the early postoperative period may lead to early detection of patients at risk of major complications allowing for alternative management strategies to improve outcomes.

## Introduction

Cytoreductive surgery (CRS) with heated intraperitoneal chemotherapy (HIPEC) as described by Sugarbaker et al. is the standard of care for peritoneal mesotheliomas and peritoneal metastasis from other organs such as the appendix, ovary, stomach, colon, and small bowel [1], [2], [3], [4], [5], [6]. Post-operative morbidity is a significant concern in patients that undergo CRS and HIPEC due to aggressive cytoreductive surgery requiring peritoneal and abdominal organ resections, toxicity due to chemotherapy, and hyperthermia achieved during perfusion of chemotherapy. Postoperative high-grade morbidity (Clavien-Dindo Grade ≥3A) and mortality following CRS and HIPEC varies widely and is proportional to the extent of cytoreductive surgery [7]. Reported morbidity and mortality outcomes after CRS and HIPEC are reported to range from 20–30 % and 1–3 %, respectively [8], [9], [10]. High-grade complications can be fatalistic and warrant early detection and treatment to improve immediate and long-term survival.

Major high-grade complications witnessed frequently after CRS and HIPEC include both infectious and non-infectious complications such as anastomotic leaks, deep space infection, pancreatitis, fistula, pulmonary embolism, renal failure, etc. [10, 11]. Use of inflammatory markers in the serum has been investigated for diagnostic and prognostic value in various types of abdominal and non-abdominal surgeries and more recently in patients who underwent CRS and HIPEC. Various inflammatory markers such as C-reactive protein (CRP), leukocyte count, neutrophil to lymphocyte ratio (NLR), procalcitonin levels, etc. have been studied in the past for early detection of the above-mentioned complications. Low cost, easy availability, non-invasiveness of the test and, promising data from previous studies were some of the factors why CRP was chosen as the inflammatory marker for our study [12]. We sought to better understand the change in CRP levels over time in the early postoperative course if they relate to major postoperative complications.

## Methods

Retrospective analysis of two prospective databases from two different institutions (Mayo Clinic, Rochester, USA and University of Vienna Medical Center, Vienna, Austria) was conducted and all patients that underwent cytoreductive surgery with HIPEC from 2011–2021 were included (n=184). Informed consent was taken from all study participants, and the study was approved by the Ethics Committee of all participating institutions. In a second step an independent database from a third institute (Barmherzige Brüder Regensburg, Germany) was analyzed for data validation (n=110). Data sharing agreement was signed between all three institutions. HIPEC regimens (drug, dosage and duration) varied not only between institutions but also within the different institutes over time and thus were not standardized.

### Inclusion criteria

Consecutive adult patients undergoing CRS with HIPEC for peritoneal carcinomatosis who had CRP levels available post-operatively.

Data from both the centers were combined for baseline characteristics that included age, sex, body mass index (BMI), smoking status, preoperative comorbidities; disease characteristics (origin of primary tumor, timing of metastasis); peri-and intra-operative factors such as anastomotic leaks, blood transfusions; major postoperative complications; and postoperative CRP levels for 7 consecutive days. Major postoperative complications were defined as Clavien-Dindo as Grade ≥3A occurring within 30 days of surgery. Anastomotic leak was defined as a defect seen in the anastomosis at reoperation, presence of feculent fluid in a pelvic drain, or evidence of free air, fluid, or extra-luminal contrast around the anastomosis on computed tomography. Absolute CRP measurements (in mg/L) on postoperative days (POD) 1–7 were compared and analyzed to find significant cut-offs that can predict all major complications. Additionally, we calculated the proportional change of CRP levels between post-operative day 1 and 4 defined as Delta (Δ) CRP POD 1/4 using following formula: ΔCRP (%) = (CRP POD 4 – CRP POD 1)/CRP POD 1 * 100. Our finding of proportional change in CRP levels between post-operative day 1 and 4 as an optimal threshold for predicting major postoperative complications was then validated in an independent German database.

### Statistical analysis

For statistical analysis IBM Statistical Package for the Social Sciences (SPSS) Version 24 for Mac (SPSS Inc., Chicago, IL, USA) was used. Descriptive data was presented as numbers (n) and percentage (%) for categorical variables and as median and interquartile range (IQR) for continuous variables. To calculate group differences for continuous variables Mann-Whitney-U-tests and Fisher’s exact test were used accordingly and χ^2^-test for categorical variables. Statistical significance was considered at a two-sided p-value ≤ 0.05. The receiver operating characteristics (ROC) curve was used to evaluate prognostic values and was reported as area under the curve with an asymptomatic 95 % confidence interval (CI). Best predictive value and cut-off values were calculated using the Youden index (YI). Negative predictive value (NPV) and positive predictive value (PPV) was calculated. Furthermore, univariable and multivariable analysis was performed with logistic regression models. In a first step, univariable analysis was performed for potential risk factors. Second, significant parameters with a p-value of <0.5 were included in a multivariable regression analysis with stepwise forward selection.

## Results

### Baseline characteristics and complications

A total of 184 patients met inclusion criteria. Baseline characteristics are outlined in Table 1. The overall major complication rate (Clavien-Dindo≥3A) was 25 % (n=46) and the anastomotic leak rate 10.9 % (n=13/119). Twenty-two patients needed re-operation (12 %) and three patients died within thirty days of the operation (1.6 %). Perioperative characteristics and their association with major complications is outlined in Table 2.

**Table 1:** Demographics and baseline characteristics (numbers and percentage, median, and IQR).

	Overall (n=184)	Major complication	Yes (n=46)	
		No (n=138)		
Sex				p=0.008^a^
Female	103 (56)	85 (61.6)	18 (39.1)	
Male	81 (44)	53 (38.4)	28 (60.9)	
BMI	24.69	24.6	24.9	p=0.982
	(22.31–28.18)	(22.3–28.3)	(21.8–27.7)	
Age, years	56	57	56	p=0.394
	(45–65)	(45–64)	(48–68)	
Current smoking				p=0.043^a^
No	169 (91.8)	130 (94.2)	39 (84.8)	
Yes	15 (8.2)	8 (5.8)	7 (15.2)	
Diabetes				p=0.490
No	172 (93.5)	128 (92.8)	44 (95.7)	
Yes	12 (6.5)	10 (7.2)	2 (4.3)	
Primary histology				p=0.286
Appendix	76 (41.3)	56 (40.6)	20 (43.5)	
CRC	45 (24.5)	35 (25.4)	10 (21.7)	
Small bowel	15 (8.2)	8 (5.8)	7 (15.2)	
Gastric	11 (6)	10 (7.2)	1 (2.2)	
Pancreas	2 (1.1)	1 (0.7)	1 (2.2)	
Gallbladder	12 (6.5)	8 (5.8)	4 (8.7)	
Peritoneum	23 (12.5)	20 (14.4)	3 (6.5)	
Timing of metastasis				p<0.001^a^
Synchronous	123 (66.8)	102 (73.9)	21 (45.7)	
Metachronous	61 (33.2)	36 (26.1)	25 (54.3)	
Neoadjuvant chemotherapy				p=0.746
No	78 (42.4)	61 (44.2)	17 (37.8)	
Yes	97 (52.7)	71 (51.4)	26 (57.8)	
Unknown	8 (4.3)	6 (4.3)	2 (4.4)	
Preoperative albumin level	4.2 (3.96–4.46)	4.2 (3.9–4.4)	4.18 (3.8–4.3)	p=0.074

^a^Significant at a p-level <0.05.

**Table 2:** Perioperative Characteristics (numbers and percentage, median, and IQR).

	Overall (n=184)	Major complication	Yes (n=46)	
		No (n=138)		
PCI	11 (4–19)	9 (4–19)	12 (5–18)	p=0.361
Completeness of cytoreduction (CC)				p=0.843
0	133 (72.3)	101 (73.2)	32 (69.6)	
0/1	27 (14.7)	20 (14.5)	7 (15.2)	
1	18 (9.8)	12 (8.7)	6 (13.0)	
2	2 (1.1)	2 (1.4)	0	
3	4 (2.2)	3 (2.2)	1 (2.2)	
Length of surgery, minutes	518	503	570	p=0.004
	(450–616)	(421–596)	(485–631)	
Gastrointestinal anastomosis				p=0.423
No	65 (35.3)	51 (37.0)	14 (30.0)	
Yes	119 (64.7)	87 (63.0)	32 (69.6)	
HIPEC agent				p=0.400
Mitomycin C	39 (21.2)	27 (19.6)	12 (26.1)	
Cisplatin	16 (8.7)	14 (10.1)	2 (4.3)	
Oxaliplatin	20 (10.9)	14 (10.1)	6 (13)	
Mitomycin/doxorubicin	74 (40.2)	60 (43.5)	14 (30.4)	
Cisplatin/doxorubicin	11 (6)	8 (5.8)	3 (6.5)	
Mitomycin/cisplatin	21 (11.4)	12 (8.7)	9 (19.6)	
Others	3 (1.5)	3 (2.1)	0	
Amount of intraoperative and postoperative packed red blood cells (PRBC) transfusion				p=0.614
0	145 (78.8)	110 (79.7)	35 (76.1)	
1	15 (8.2)	11 (8.0)	4 (8.7)	
2	11 (6.0)	6 (4.3)	5 (10.9)	
3	2 (1.1)	2 (1.4)	0	
>3	4 (2.2)	3 (2.1)	1 (2.2)	
ICU stay				p=0.667
No	79 (42.9)	58 (42.0)	21 (45.7)	
Yes	105 (57.1)	80 (58.0)	25 (54.3)	
Length of ICU stay	2 (1–3)	2 (1–2)	3 (1–8)	p=0.009
Length of hospital stay	9 (7–14)	9 (7–13)	17 (8–28)	p<0.001

In univariate analysis female sex (p=0.008), active nicotine abuse (p=0.043), longer operating time (p=0.004), and metachronous metastasis (p<0.001) were all significantly associated with an increased risk of major complications postoperatively.

As expected, patients who experienced a major postoperative complication had not only a significantly longer stay in the ICU (p=0.009) but also in the hospital (p<0.001).

### CRP levels and major postoperative complications

**Figure 1:**
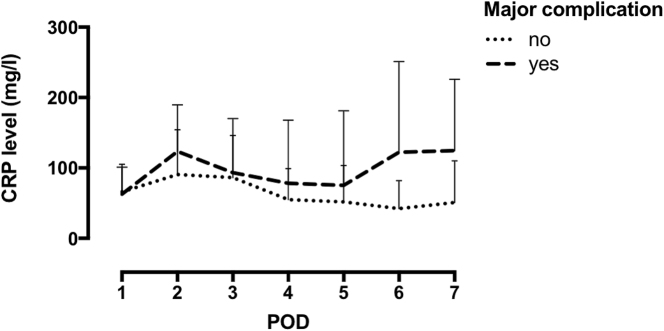
Rise and fall of C-reactive protein postoperatively [* statistically significant on POD 4 and POD 6].

First, we evaluated the dynamic of CRP levels comparing patients with and without major complications which showed higher CRP levels in the patients who had post-operative complications as compared to the patients that did not. In our cohort 25 % (n=46) of all patients developed a major complication (Clavien-Dindo Grade ≥ 3A) postoperatively. We found significantly higher CRP levels for patients developing major complications on postoperative day (POD) 2 and from POD 4 onwards (Figure 1).

**Figure 2:**
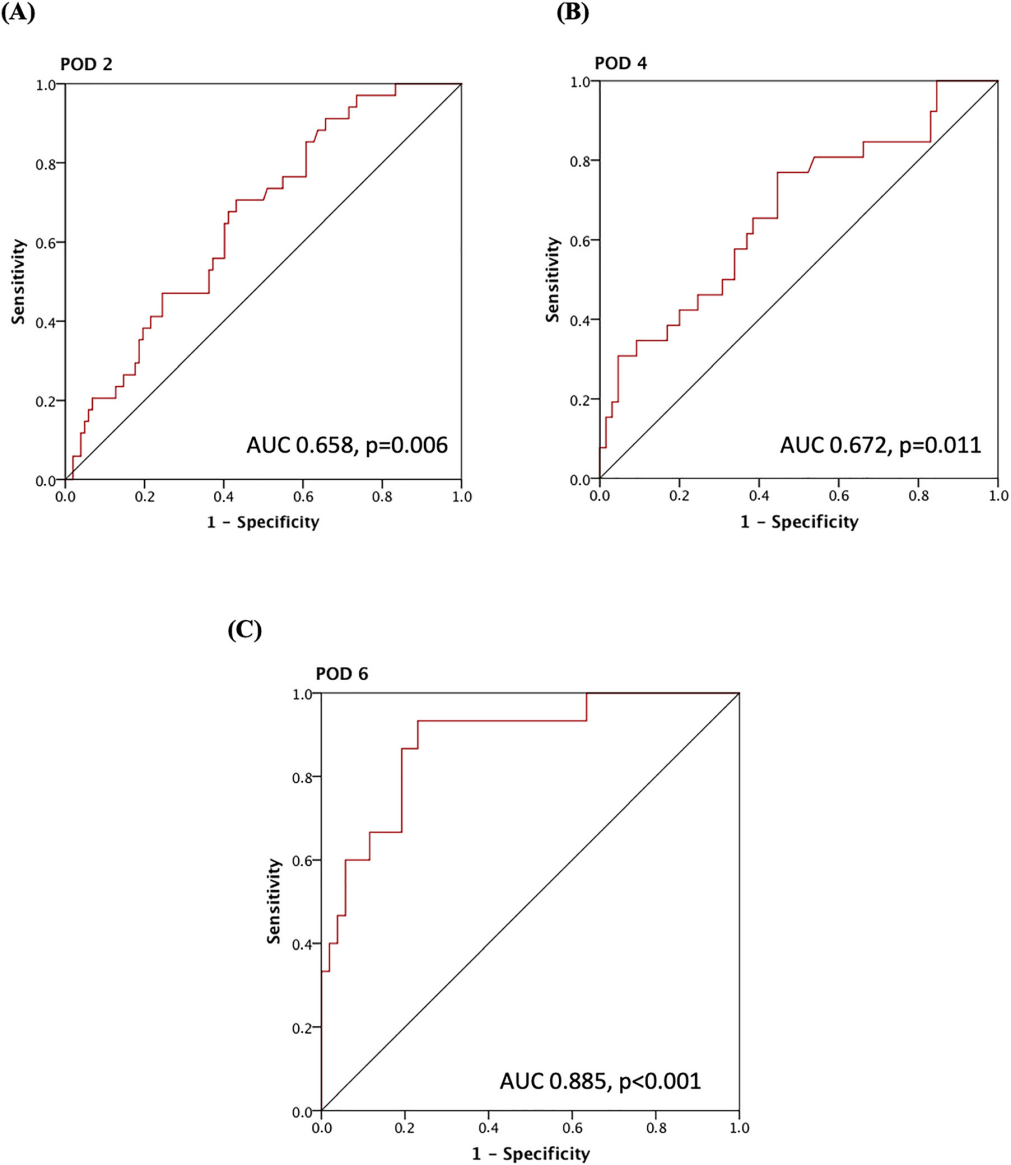
Different ROC-Curves for CRP levels and postoperative complications on (A) POD 2 (n=136). (B**)** POD 4 (n=91), and (C) POD 6 (n=67).

In a next step, we assessed the potential of CRP levels to predict major postoperative complications. The predictive value of CRP for major postoperative complications showed an AUC of 0.658 on POD 2 (p=0.006, 95 % CI 0.56–0.76) in the ROC curve (Figure 2A). The cut-off of 106 had a sensitivity of 67.6 % with specificity 58.8. PPV 35.4 %, NPV 84.5 %. On POD 4 the AUC was 0.672 (p=0.011, 95 % CI 0.55–0.78). The cut-off of 65.5 had a sensitivity 76.9 % and specificity 55.4 %, PPV 40.8 %, NPV 85.7 % (Figure 2B).

The predictive value of CRP increased even more on POD 6 with an AUC of 0.885 (p<0.001, 95 %CI 0.79–0.98). A cut-off of 74 had a sensitivity of 93.3 %, specificity 76.9 %, PPV 53.8 %, and NPV 97.6 % for major complications (Figure 2C).

### Delta CRP as an early predictor for major postoperative complications

As an early predictive value for major postoperative complications we evaluated the predictive value of the CRP dynamic from POD 1 to POD 4.

Definition: ΔCRP (%) = (CRP POD 4 – CRP POD 1)/CRP POD 1 * 100.

**Figure 3:**
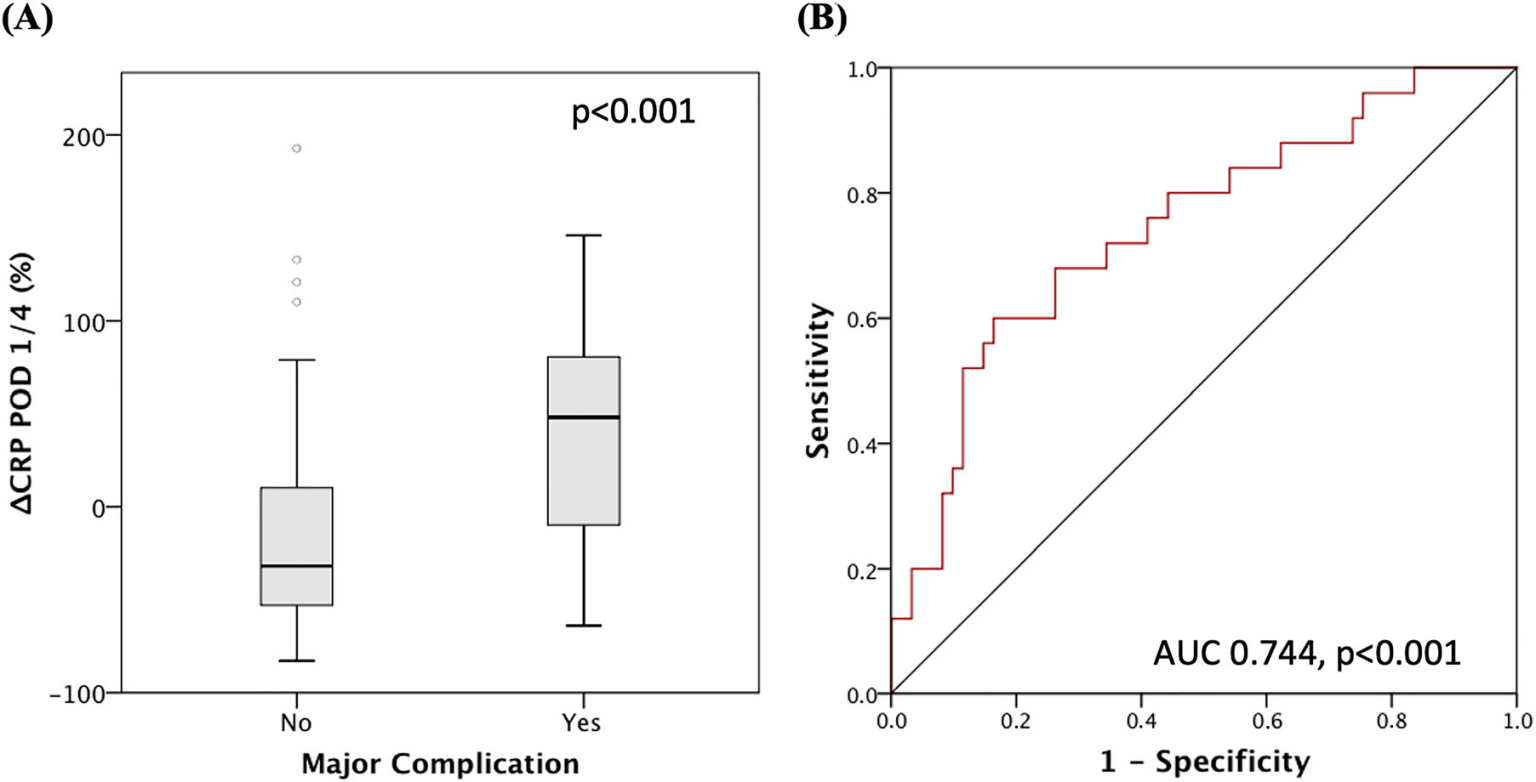
(A) Proportional change of CRP level from POD 1 to 4 for patients with compared to patients without complications. (B) ROC-curve for predictive value of ΔCRP POD 1/4 for the development of a major postoperative complication.

We saw a significant difference in the proportional change of CRP levels from POD 1 to POD 4 between patients with major and those without major complications [p<0.001, 48.3 (IQR-14.1–91.0) vs. −31.7 (IQR-53.7–11.3), Figure 3A].

The predictive value of ΔCRP POD 1/4 expressed as the AUC was 0.744 (p<0.001, 95 % CI 0.628–0.861). In the ROC curve a cut-off of 30 % was calculated with a sensitivity of 60 % and specificity of 83.6 % for major complications (Figure 3B). The NPV is 83.6 % and PPV is 60 %.

To assess whether ΔCRP (POD 1/4) is independently associated with postoperative complications we included potential risk factors for postoperative complications in a univariable regression models. Sex, smoking habit, timing of metastasis, length of surgery, CRP levels on POD 2 and 4, and postoperative dynamic from POD 1 to 4 at a cut-off of 30 % were included for multivariable analysis. While female sex, active nicotine abuse, longer operative time, and metachronous disease presentation were statistically significant on univariate analysis only ΔCRP POD 1/4 at a cut-off of 30 % (Δ CRP^high^ vs. Δ CRP^low^) remained as an independent risk factor for major complications in the multivariate analysis (Table 3).

**Table 3:** Univariable and multivariable analysis for major complications.

	Univariable	Multivariable	95 % CI	p	OR	95 % CI		
	p	OR						
Sex	0.009	0.401	0.202	0.795				
BMI	0.996	1.000	0.933	1.072				
Age	0.260	1.015	0.989	1.041				
Current smoking	0.051	0.343	0.117	1.005				
Diabetes	0.495	1.719	0.362	8.149				
Timing of metastasis	0.001	0.296	0.148	0.593				
Neoadjuvant therapy	0.445	0.761	0.378	1.533				
Preoperative albumin level	0.032	0.567	0.338	0.953				
PCI	0.397	1.016	0.979	1.055				
CCR	0.734	1.066	0.737	1.543				
Length of surgery	0.015	1.003	1.001	1.006				
GI anastomosis	0.424	0.746	0.364	1.528				
Postoperative PRBC	0.405	0.700	0.303	1.619				
CRP POD 2	0.012	1.008	1.002	1.013				
CRP POD 4	0.004	1.012	1.004	1.021				
Δ CRP^high^/Δ CRP^low^ ^a^	0.001	0.131	0.046	0.373	0.001	0.086	0.025	0.293

^a^Significant at a p-level <0.05.

### Validation of delta CRP as a predictive marker for major complications in an independent cohort

In a next step we externally validated our findings in an independent cohort. We therefore evaluated the predictive value of ΔCRP POD 1/4 and the cut-off of 30 % in a similar patient cohort from a German HIPEC Center, Barmherzige Brüder Regensburg. The cohort consisted of all patients that had CRS and HIPEC in 2018 (n=110).

**Figure 4:**
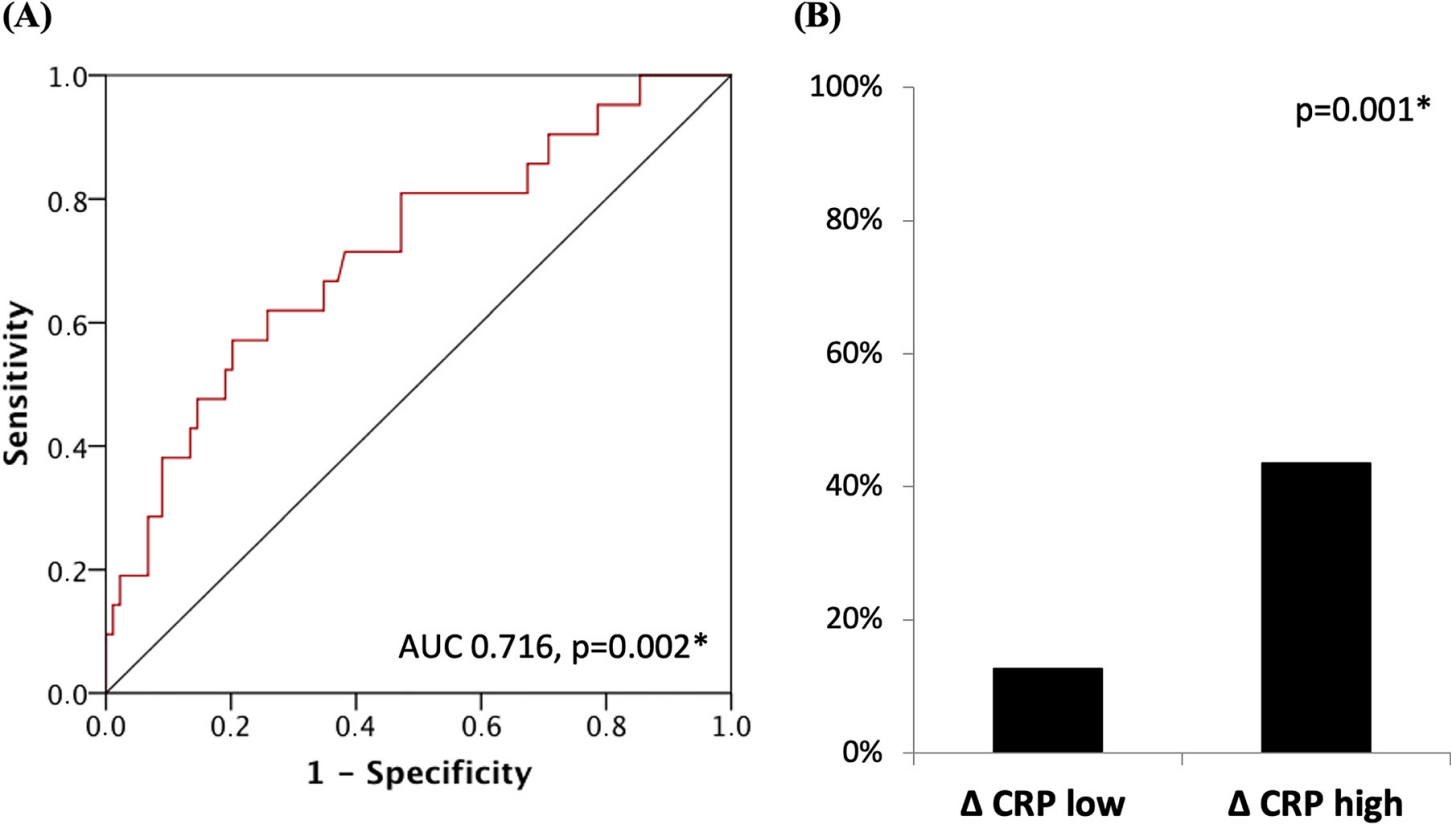
(A) Shows the ROC-Curve for predictive value of ΔCRP POD 1/4 for the development of a major postoperative complication in an independent external validation cohort. (B) Shows the percentage of major complication in the two different risk groups (Δ CRP^low^ vs. Δ CRP^high^).

The predictive value of ΔCRP POD 1/4 expressed as the AUC was 0.716 (p=0.002, 95 % CI 0.590–0.842) (Figure 4A). The cut-off of 30 % was significantly associated with major complications (p=0.001, 11 of 87 [12,6 %] in Δ CRP^low^ vs. 10 of 23 [43.5 %] in Δ CRP^high^) (Figure 4B).

### Delta CRP and the prediction of unplanned re-operation

Furthermore, we did a subgroup analysis of patients who needed re-operation. In the entire cohort 34 patients (11.5 %) needed re-operation within the first 30 days postoperatively. ΔCRP POD 1/4 was significantly different for patients who had to undergo surgical interventions postoperatively (p<0.001). The predictive value of ΔCRP POD 1/4 expressed as the AUC was 0.791 (p<0.001, CI 0.706–0.876). The cut-off of 7 % showed a sensitivity of 70.4 % and a specificity of 75.1 %. The NPV was 94.1 % and the positive predictive value 16.4 %.

## Discussion

In our study we found a proportional increase of CRP from the first to fourth postoperative day of more than 30 % to be a significant predictor for major postoperative complications within 30 days of index operation. This relative value may overcome limitations of institutional differences in absolute CRP cut-off values and CRP variability due to other reasons.

C-reactive protein is an important systemic inflammatory biomarker that has been evaluated for predicting postoperative complications after several major oncologic operations [13], [14], [15], [16], [17], [18]. One common finding of recent studies evaluating postoperative predictive factors of complications is that CRP is a more sensitive predictor of severe complications than leukocytosis. This is likely due to the bone marrow suppression seen with mitomycin C and other myeloablative HIPEC agents. The common need for splenectomy further lessens the sensitivity and specificity of leukocytosis in predicting postoperative complications. Thus, an inflammatory biomarker unaffected by the chemotherapy or organs resected is an unmet need.

The diagnostic accuracy of CRP for predicting major complications is important for both identifying high risk patients for possible early diagnostic and management changes as well as identifying low risk patients on increasing enhanced recovery after surgery (ERAS) protocols for early discharge and return home. Several recent publications have specifically investigated postoperative CRP values after cytoreductive surgery and HIPEC. Different studies have identified different cut-off values on different days for predicting major postoperative complication. Similarly, we found several significant cut-off values. On POD 2 a cut-off of 106 mg/L has a sensitivity of 67.6 % and an NPV of 85.7 % (p=0.006) for predicting major complications. The sensitivity increases to 76.9 % on POD 4 with a cut-off of 65.5 mg/L. The sensitivity and NPV increase furthermore on POD 6, but this may be too late for recognition of post-operative complications. Unfortunately, the cut-off values from different studies show a great variation. Compared to other studies evaluating CRS with and without HIPEC, our absolute CRP cut-offs are fairly low for predicting complications (186.1 on POD 2 by Gans et al.; mean CRP of 162.4 between POD 2 and 4 by Asmar et al.; 166 on POD 3 and 116 on POD 4 by Kooten et al.) [16, 17, 19]. Even though baseline and treatment characteristics seem to be similar to these studies some differences according to indication, extend of surgery or risk constellation that influence CRP levels may not be reflected in these publications.

The discrepancy may also reflect the differences in CRP assays across the globe or a difference in baseline CRP levels as CRP levels appear to differ across patients, neoadjuvant therapies and tumor histologies [20], [21], [22]. Unfortunately, we did not have baseline preoperative CRP levels available for this study. In fact, when comparing our results with previously published studies a significant institutional variability is clearly visible between cut-offs. Our study is more generalizable as this is the largest study to date; the patient population included is from two different institutions located on two different continents with very different histologies and perioperative care. However due to these institutional differences in CRP assays, surgical and perioperative care, we hypothesize that it will not be feasible to find a single absolute CRP cut-off that can predict the probability of occurrence of major complications for all institutions around the globe. Therefore, it would be necessary to determine their own unique cut-off for predicting postoperative complications.

To address this issue, we discovered that CRP dynamic represented as the ‘proportional change in CRP- ΔCRP’ can significantly predict the development of all major complications. Past studies have shown both increase and decrease in CRP levels with the type of anesthetic agents used during the surgery, or with the post-operative analgesics, anti-hypertensives, beta-blockers etc. [23], [24], [25], [26], [27]. As this variable represents a proportional change we can hypothesize that it is less affected by the differences in operative and perioperative care. A similar but not same variable has previously been studied for detecting post-bariatric surgery complications by Duprée et al. and for predicting anastomotic leaks after colorectal anastomosis by Stephensen et al. [28, 29]. Proportional change in CRP between POD 1 and POD 4 at a cut-off of 30 % has a sensitivity of 60 % and specificity of 83.6 % (p<0.001) for predicting major complications and is an independent risk factor for major complications (Table 3). As ΔCRP is a relative value over time, the ability to predict both infectious and non-infectious complications independent of factors such as different HIPEC protocols is a major strength of our study. ΔCRP was then independently validated in a third independent dataset from a German center that was statistically significant with an AUC of 0.716 (p=0.002, 95 % CI 0.590–0.842) (Figure 4A, 4B). Furthermore, the proportional change of CRP may also serve as a role out parameter for patients that may have to undergo re-operation as we found that a cut-off of 7 % has a negative predictive value of 94.1 %.

## Conclusions

Increasing evidence suggest that postoperative CRP levels can identify patients that are at risk for development of major complications following CRS and HIPEC, leading to further workup and alteration of management strategies. However, absolute CRP cut-off levels have varied across studies possibly due to different assays, techniques, equipment, baseline levels, and peri-operative care. The delta CRP may compensate for these variances and provide the best discernment for the development of major complications in the immediate postoperative period and can aid in early diagnosis and management. This was further externally validated.

### Limitations

The retrospective nature of the study design, limited sample size, incomplete postoperative CRP data, and the heterogeneity in surgical and perioperative care practice were some of the limitations of the study which may have introduced bias and limited analysis. We also were not able to account for other factors such as the type of anesthesia, or perioperative anti-inflammatory agents (steroids, NSAIDs, etc.) had any impact on postoperative outcome, or on CRP itself. However, these same limitations also give this study more generalizability as the cohort was very heterogenous in terms of histology, and perioperative care.
